# Thermodynamic Performance Analysis of a Waste Heat Power Generation System (WHPGS) Applied to the Sidewalls of Aluminum Reduction Cells

**DOI:** 10.3390/e22111279

**Published:** 2020-11-11

**Authors:** Yong Ming, Naijun Zhou

**Affiliations:** School of Energy Science and Engineering, Central South University, Changsha 410083, China; mingyong@csu.edu.cn

**Keywords:** aluminum reduction cells, waste heat power generation, Organic Rankine Cycle, thermodynamics analysis

## Abstract

To recover energy from the waste heat of aluminum reduction cells, a waste heat power generation system (WHPGS) with low boiling point working fluid based on Organic Rankine Cycle was proposed. A simplified model for the heat transfer around the walls of aluminum reduction cells and thermodynamic cycle was established. By using the model developed and coded in Matlab, thermal performance analysis of the system was conducted. Results show that the electrolyte temperature and the freeze ledge thickness in the cell can significantly affect the heat absorption of the working fluid in the heat exchange system on the walls. Besides, both the output power and the thermal efficiency of the power generation system increase with the system pressure. The output power and thermal efficiency of the system can also be affected by the type of working fluid used in the system. Working fluids for the best system performance under different output pressures were determined, based on the performance analysis. This WHPGS would be a good solution of energy-saving in aluminum electrolysis enterprises.

## 1. Introduction

The electrolytic aluminum industry is the industry of high energy consumption. Nowadays, the annual production of electrolytic aluminum is close to 40 million tons in China. The production of primary aluminum in 2019 was 36.487 million tons. The electricity energy consumption of the electrolytic aluminum industry accounted for about 10% of the domestic gross power generation. The energy efficiency of aluminum reduction cells is usually less than 45%, and thus a big part of the energy, usually accounting for about half of the system heat dissipation, is discharged into the environment from the side wall of the electrolytic cells in the form of heat. However, as a low-grade energy, it is difficult to be effectively used.

Some Methods have been proposed for the recovery and utilization of the residual heat of the sidewall. Siljan [1] used a silicon carbide forming cooling device on the inner side of the steel cell to recover the heat. The cooling fluid was gas and liquid. Gas could be air, N2, Ar, and He, etc., and liquid could be molten salt and heat oil, etc. Ingo Bayer et al. [2] installed a heat pipe between the silicon carbide plate in the cell side and the shell, and the tube was filled with air or liquid fluid so that the heat could be used to preheat aluminum oxide powder. Wang et al. [3] designed a new type of reduction cell. The heat exchanger was installed inside the cell, and heat was transferred and taken out by the molten salt. Zhang et al. [4] set up a semiconductor thermoelectric generation device in the outer shell of the electrolytic cell to generate electricity directly. Liang [5] recovered the aluminum reduction cell’s sidewalls waste heat based on thermoelectric conversion. The result showed that the temperature difference of the thermoelectric generator can reach 100 °C, the output power of the waste heat generating system made up of 18 thermoelectric generator is 290 W, and the output power was poor. Due to the drawbacks existing in the above methods, none of them have been widely used.

The power generation system based on Organic Rankine Cycle with low boiling point refrigerant can effectively recover energy from low-temperature waste heat. With other advantages such as simple equipment requirement, no pollution, etc., it has gained the interest of some research groups. For example, Ma et al. [6] found that R717 achieved the best performance when the heat source temperature is less than 120 °C, and methanol is the optimal working fluid when the heat source temperature is between 160~200 °C. Bellos et al. [7] presented a hybrid Organic Rankine Cycle (ORC) driven by solar energy and waste heat. Parabolic trough collectors coupled to a storage tank feed the heat recovery system, which also utilizes waste heat of low-grade temperature (150–300 °C). Four different working fluids (toluene, cyclohexane, MDM (octamethyl trisiloxane), and n-pentane) are examined in the regenerative ORC. The results showed that toluene leads to the highest electricity production, with cyclohexane, MDM, and n-pentane to follow. Li et al. [8] used a comprehensive evaluation criterion combined economic performance and exergy efficiency for the working fluids’ optimization of ORC. In the ranges of evaporating temperature and condensing temperature, R113 and R245ca present better economic performance than other work fluids, while the exergy efficiency of R161 is highest. The comprehensive evaluation function shows that the R161 has the best working performance. Shen et al. [9] reveals both system topological structure and the component characteristics, but also connects the system output to the boundary conditions, the structural parameters, and the operating parameters. Operation optimizations for a certain ORC system are implemented via genetic algorithm (GA). The results show that with a 16 K increment of heat source temperature, the net power output and thermal efficiency will increase by 84% and 25%, respectively. On the other hand, the heat source flow rate shows limited impact on optimizations. Sun et al. [10] found that the relationships between controlled variables (optimal relative working fluid mass flow rate, the optimal relative condenser fan air mass flow rate) and uncontrolled variables (the heat source temperature and the ambient dry bulb temperature) are near liner function for maximizing system net power generation and quadratic function for maximizing the system thermal efficiency. Roy et al. [11] compared the system and second law efficiency, irreversibility of the system, availability ratio, work output, and mass flow rate with increasing turbine inlet temperature (TIT) under different heat source temperature conditions. Roy et al. [12] carried out parametric optimization and performance analysis for a regenerative Organic Rankine Cycle using R123 applied to waste heat recovery. Wang et al. [13] studied the working fluid and performance of a power generation system applied to low-temperature waste heat by using the simulated annealing algorithm. Jankowski et al. [14], in order to determine which one of the considered criteria dominates significantly, conducted multi-objective optimization using weight function G and genetic algorithm (GA). The analysis was carried out for three sampled working fluids of the ORC: R1234yf, R1234ze, and R245fa. The final results showed that R1234yf was the optimal choice, since, in case of that fluid, the lowest values of the function G were observed for all weight distribution cases. Sanchez et al. [15] have investigated the use of binary zeotropic mixtures as working fluids applied to Organic Rankine Cycles (ORCs). The mixtures were tested with a basic Rankine cycle while using the heat source temperature as an independent variable, which assumed six different values, ranging from 80 to 180 °C, in steps of 20 °C. The results showed that the range of the so-called ideal mixtures narrows as the heat source temperature increases, with mixtures including fluids like R245fa and pentane being good options, whereas at low temperature, a larger number of fluid mixtures perform well. Xu et al. [16] applied linear Fresnel reflector solar concentrator (LFR) to absorbing solar energy and constructed an ORC system, and the result shows that cyclohexane is the preferred working fluid. Ge et al. [17] presented a dual-loop Organic Rankine Cycle (DORC). Cyclopentane/cyclohexane and benzene/toluene mixtures are used for high-temperature loop (HTL), whereas isobutane/isopentane (R600a/R601a) mixtures are selected as working fluids for low-temperature loop (LTL). The influences of engine exhaust gas temperature on net power output and exergy destruction rate are also discussed. Results show that the use of mixtures for the two loops can reduce the exergy destruction rate of HTL evaporator and LTL condenser compared to that of a pure working fluids system and the exergy destruction rate of condenser/evaporator compared to that of mixtures using only a one loop system. Wang et al. [18] analyzed the performance of ORC to recover low-temperature gas waste from an aluminum reduction cell. The results showed that there was optimal evaporating temperature for maximum net power under the same pinch point. For a heat source temperature range of 80~140 °C and 150~170 °C, the working fluids given the biggest net power are R227ea and R236fa, respectively. With the development of low-temperature electrolysis technology, the exhaust gas temperature is getting lower and lower and the utilization value is very small, while the sidewalls temperature is between 200~300 °C and has a good utilization prospect.

Generally speaking, researchers have done some quotable work on the working fluid selection and system optimization of ORC. However, there is no report yet on the application of an ORC power generation system used for recovering heat of the aluminum reduction cell’s sidewalls. Considering the features of the heat dissipation of the aluminum reduction cell’s wall, a WHPGS based on flash cycle with organic working fluids was proposed. A thermodynamic calculation model of flash cycle with low-boiling working fluids and a simplified calculation model for the heat exchange system of the cell’s wall were established and used to analyze the thermal performance of the system.

## 2. System Description

**Figure 1:**
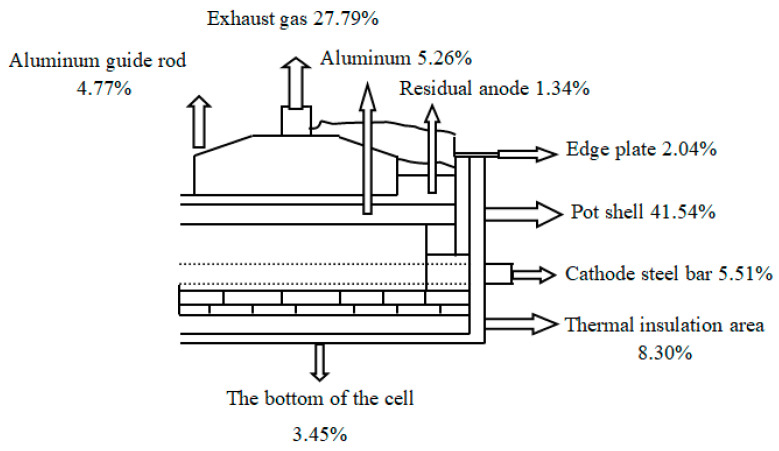
Structure diagram of prebaked cell.

**Figure 2:**
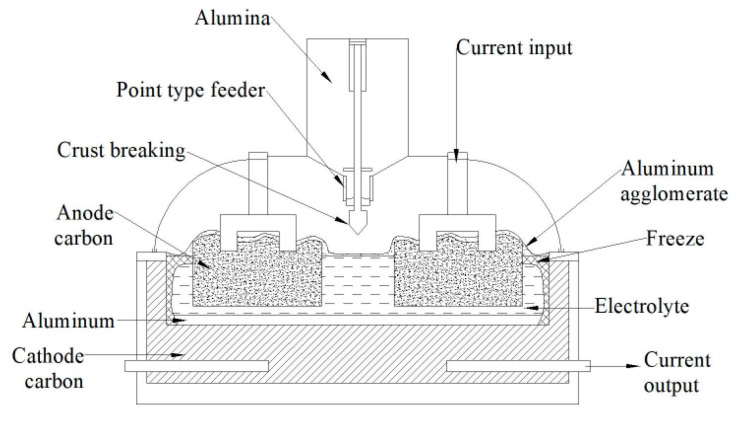
Heat dissipation distribution of 200 kA cell.

The reduction cell is the main equipment of aluminum smelting, and the typical prebaked aluminum reduction cell structure is shown in Figure 1. The basic process of aluminum smelting is as follows: Alumina melts in cryolite, under the action of current, CO_2_ and CO are produced on the anode carbon, and the molten aluminum is deposited at the bottom of the cathode carbon. In this process, some heat is used to heat the material in the cell to the electrolysis temperature and to complete the electrolysis reaction at this temperature, while the rest is lost to the surrounding environment in the form of heat. A typical 200 kA cell heat dissipation distribution is shown in Figure 2.

**Figure 3:**
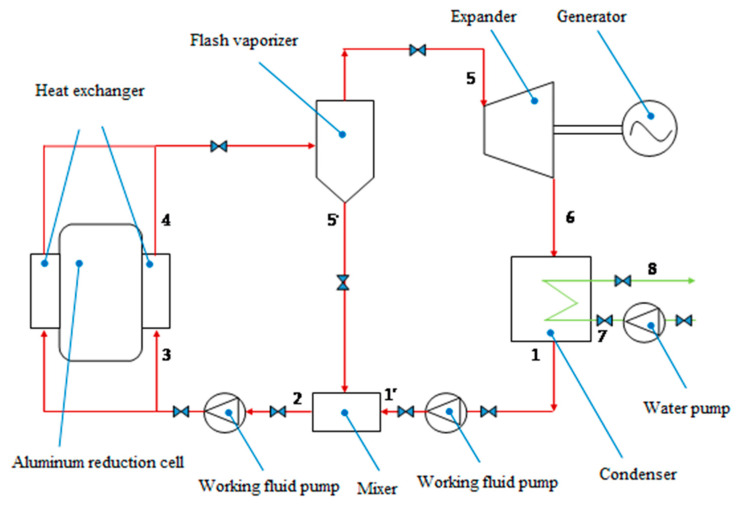
Schematic diagram of the waste heat power generation system (WHPGS).

A WHPGS applied for the sidewall of aluminum reduction cells based on flash cycle with organic working fluids was proposed in this work. The schematic diagram is shown in Figure 3. The system consists of a group of heat transfer units installed at the sidewalls of aluminum reduction cells, a flash evaporator, a mixer, an expander with low boiling point fluid, a generator set, a condenser, and two working fluid pumps, etc. The digits in the figure represent the state codes of the import and export working fluid of each part.

In the system, a group of transfer units are installed in the ventilation space of an aluminum reduction cell, and the working fluid is heated into saturated liquid state by the heat released from the aluminum reduction cell’s shells (the reason for being heated into saturated liquid state instead of saturated gas state is that the space for the transfer unit is too narrow, has low efficiency of heat transfer and has low operating reliability). Liquid working fluid flows into the flash evaporator, where some of the fluids become saturated vapor, and the other fluids flow into the mixer, mix with the condensed fluid outside the condenser outlet, and then go back to the transfer unit through the feed pipe. The saturated vapor comes out of the flash evaporator and enters the expander for work, while driving the generator to generate electricity simultaneously. The exhaust steam after expansion flows into the plate-type condenser and becomes liquid due to condensation by recirculating cooling water, and then is transferred to the mixer by the working fluid pump.

By taking advantaging of the Organic Rankine Cycle, waste heat from walls of aluminum reduction cells can be converted into high-quality electricity by this system with higher energy conversion efficiency, flexible layout installation, and good practical value. Using this system, energy-saving technical reconstruction of existing aluminum reduction cells of different capacities can be realized, which basically does not affect the original production operation when operating, and the risk of reconstruction is also negligible.

## 3. The Model for the Heat Transfer System

**Figure 4:**
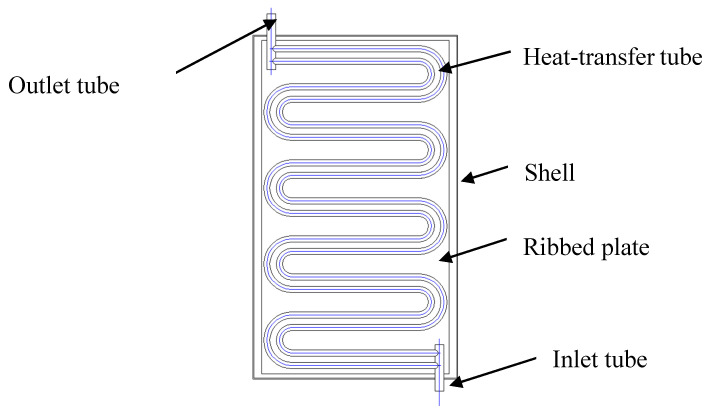
Structure of a wall heat exchanger.

To obtain the waste heat from sidewalls of aluminum reduction cells, the heat exchanger was designed as shown in Figure 4, and main parameters are shown in Table 1. In the heat exchanger, heat-transfer tubes are made of copper with a serpentine arrangement. The aluminum reduction cell was charged. In order to ensure the safe operation of the system, we need something that has good thermal conductivity and poor electrically conductivity. The heat transfer efficient and electric resistance of alumina power is 10 W/mk and 10 MΩ/KV, respectively. The heat-transfer tubes are filled with working fluid, and alumina powders with good thermal conductivity and insulation performance are padded outside the tubes. The entire heat exchanger is installed in the ventilation space of aluminum reduction cells, in a good contact with the sidewalls of the aluminum reduction cell, and an insulation wool blanket is pasted to its outer surface.

**Figure 5:**
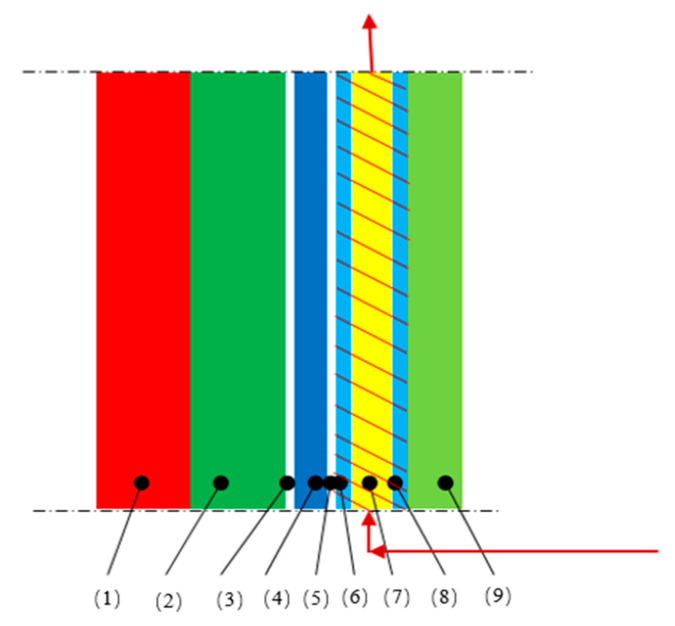
Schematic diagram of heat transfer system close to the sidewall. (1) Freeze layer, (2) Carbon block, (3) Contact layer, (4) Steel shell, (5), Contact layer, (6), Inner wall of the heat exchanger, (7) Flow channel, (8) Outer wall of the heat exchanger, (9) Insulation layer.

Considering the nonuniformity of heat exchange while the working fluid flows in the tube, the serpentine tube was divided into several parts in this work. Each part was unfolded like flat walls, and coupling calculation was made to every thermal conduction layer near the side wall of the aluminum reduction cell, and then the entire heat exchange system was simplified to a heat transfer process with one-dimensional steady state. In order to improve the accuracy of calculation, the heat transfer system was divided into several layers for successive iteration calculation, which is shown in Figure 5.

The computational method of the heat transfer system is as follows.

Corresponding to each section of the heat transfer tube, according to the energy balance equation, the following equation can be derived:

(1) $$\frac{T_{M}-T_{exi}}{R_{in}}-\frac{T_{exi}-T_{air}}{R_{out}}=\frac{{\dot{m}}_{2}C_{pi}\cdot (t_{i}-t_{i-1})}{\pi d\cdot l}$$

where $R_{in}$ is the total thermal resistance from inner freeze to working fluid, $R_{out}$ is the total thermal resistance from working fluid to circumstance, $T_{M}$ is the temperature of electrolyte in the cell, $T_{exi}$ is the temperature of working fluid in the tube, $T_{air}$ is environmental temperature outside the heat exchanger, ${\dot{m}}_{2}$ is mass flow rate of the working fluid, $C_{pi}$ is specific heat capacity of the working fluid, $t_{i-1}$ and $t_{i}$ are inlet and outlet temperatures of the working fluid in the tube, d is diameter of the tube, and l is length of every section of the tube. While, the thermal resistance can be written as:

(2) $$R_{in}=\frac{1}{h_{M}}+\sum \frac{B_{Fr}(i)}{\lambda_{Fr}(i)}+\sum \frac{B_{Ca}(j)}{\lambda_{Ca}(j)}+R_{C1}+\sum \frac{B_{St}(k)}{\lambda_{St}(k)}+R_{C2}+\sum \frac{B_{St}(l)}{\lambda_{St}(l)}+\sum \frac{B_{A}(m)}{\lambda_{A}(m)}+\sum \frac{B_{Co}(n)}{\lambda_{Co}(n)}+\frac{1}{h_{f}}$$

(3) $$R_{out}=\frac{1}{h_{f}}+\sum \frac{B_{Co}(p)}{\lambda_{Co}(p)}+\sum \frac{B_{A}(q)}{\lambda_{A}(q)}+\sum \frac{B_{st}(r)}{\lambda_{st}(r)}+\sum \frac{B_{ins}(s)}{\lambda_{ins}(s)}+\frac{1}{h_{air}}$$

where *B* is the thickness of the every layer, λ is thermal conductivity, $R_{C1}$ and $R_{C2}$ are both thermal contact resistance, h is convective coefficient, *i*, *j*, *k*, *l*, *m*, *n*, *p*, *q*, *r*, and *s* are number of temperature nodes in each layer, and Fr, Ca, St, A, Co, ins, f, and air refer to the freeze layer, carbon block, alumina, copper tube, insulating layer, working fluid, and external environment, respectively. $\lambda_{Fr}(i)$, $\lambda_{Car}(j)$, $\lambda_{St}(k)$, and $\lambda_{ins}(q)$ are thermal conductivities of every material respectively, and their values can be obtained from the test values in some literature, for example, [18,19]:

$$\lambda_{Fr}(i)=(0.825+0.550\times 10^{-3}(T(i)-273-300))\times 4.187/3.6$$

$$\lambda_{Car}(j)=(3.05+4.28\times 10^{-3}(T(j)-273-300))\times 4.187/3.6$$

$$\lambda_{St}(k)=(40.4-0.0545\times (T(k)-273-200))\times 4.187/3.6$$

$$\lambda_{ins}(s)=(0.0674+0.000215\times (T(s)-273))$$

The convective coefficient of the working fluid in each tube section was derived from Gnilinski [20]:

(4) Nufi=0.012⋅(Refi0.87−280)⋅Prfi0.4⋅[1+(di/li)2/3]⋅(Prfi/Prwi)0.11

where $d_{i}$ is inner diameter of the tube, $l_{i}$ is the length of each tube section, Refi is Reynolds number in flow, and ${\mathrm{Pr}}_{\mathrm{fi}}$ and ${\mathrm{Pr}}_{\mathrm{wi}}$ correspond to Prandtl numbers in the average temperature of fluid and wall. Therefore, for any tube section, the node temperature of each layer can be obtained by recursion with the following equations:

(5) $$T(a+1)=T(a)-(T_{M}-T_{exi})\frac{B_{x}(a)}{R_{in}\lambda_{x}(a)}$$

(6) $$T(a+1)=T(a)-(T_{M}-T_{exi})\frac{B_{x}(a)}{R_{in}\lambda_{x}(a)}$$

Equation (5) is recurrence temperature relations from the electrolyte to the working fluid. Equation (6) is recurrence temperature relations from the working fluid to the external environment. In the formula, *a* and *b* are the number of temperature nodes in each layer. *x* corresponds to an arbitrary structure layer.

After obtaining node temperature of each layer in the steady-state heat-transfer case, the surface heat fluxes can be calculated by the following equation:

(7) $${\dot{q}}_{W}=\frac{T_{M}-T_{exi}}{R_{in}}$$

where $T_{M}$ is the temperature of electrolyte in the cell, and $T_{W}$ is the temperature of the wall of the aluminum reduction cell.

## 4. The Model for the Thermodynamic Cycle System

**Figure 6:**
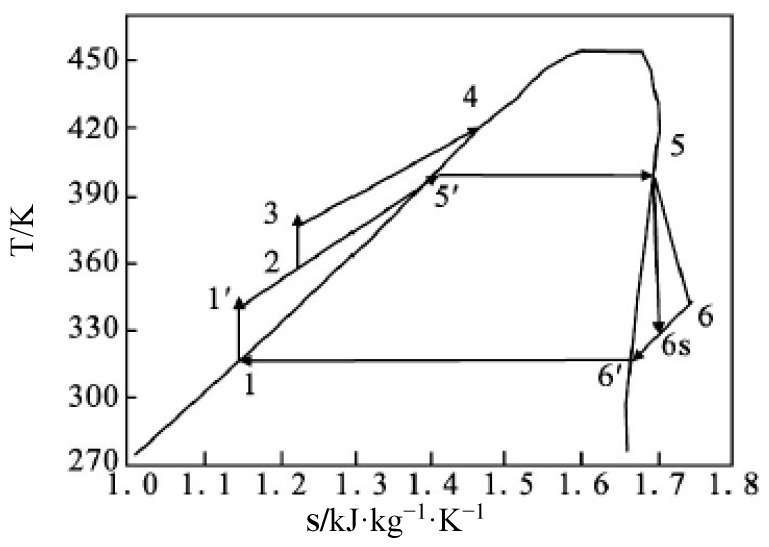
T-S (Temperature entropy) diagram of the thermodynamic process.

Combining with the system schematic diagram (Figure 3), the T-S (Temperature entropy) diagram of the thermodynamic cycle is shown in Figure 6. Compared to the conventional ORC power generation system, there is an additional flasher to complete the process of depressing flash from saturated liquid to saturated steam and an additional mixer to complete the mixture of sub-cooled liquid and the liquid of cooler outlet in the proposed ORC.

The thermodynamic processes of the cycle are included as:

1−1′: Compressing process of the fluid pump. The work of the pump is:

(8) $${\dot{W}}_{P1}={\dot{m}}_{1}(h_{1^{\prime }}-h_{1})$$

1−2: Mixing process. Regardless of the heat loss of the mixer, we have:

(9) $${\dot{m}}_{2}h_{2}={\dot{m}}_{1}h_{1}+{\dot{m}}_{5^{\prime }}^{}h_{5^{\prime }}^{}$$

where ${\dot{m}}_{1}$ is the mass flow rate of the working fluid at the condenser outlet, ${\dot{m}}_{5^{\prime }}$ is the mass flow rate of liquid at the flasher outlet, ${\dot{m}}_{2}$ is the total mass flow rate of the mixed working fluid, and $h_{1}$, $h_{5^{\prime }}$, and $h_{2}$ are the enthalpy values-related state points.

2−3: Compressing process of mixed working fluid liquid by pump. The work of the pump is:

(10) $${\dot{W}}_{P}={\dot{m}}_{2}(h_{3}-h_{2})$$

where $h_{2}$ and $h_{3}$ are the enthalpy of working fluid at the pump inlet and outlet.

3−4: Heat-absorbing process of working fluid at constant pressure. The absorbed heat is:

(11) $${\dot{Q}}_{W}={\dot{m}}_{2}(h_{4}-h_{3})$$

where $h_{3}$ and $h_{4}$ are the enthalpy of working fluid at the heat exchanger inlet and outlet.

4−(5′)−5: Flashing process. Regardless of the heat loss of the flasher, we have:

(12) $${\dot{m}}_{4}h_{4}={\dot{m}}_{5}h_{5}+{\dot{m}}_{5^{\prime }}^{}h_{5^{\prime }}^{}$$

The circulating rate is defined as:

(13) $$\phi =1/(1-\frac{{\dot{m}}_{5^{\prime }}^{}}{{\dot{m}}_{4}})$$

5−6: Work process of high-pressure steam in the expander. The efficiency of the expander is defined as:

(14) $$\phi =1/(1-\frac{{\dot{m}}_{5^{\prime }}^{}}{{\dot{m}}_{4}})$$

where $h_{5}$ and $h_{6}$ are the enthalpy of working fluid at the expander inlet and outlet, and $h_{6s}$ is the calculated enthalpy at the expander outlet based on the hypothesis that the gas is expanded at a constant entropy process. So, $\left(h_{5}-h_{6s}\right)$ is the ideal enthalpy drop of the expander. The expansion work is:

(15) $${\dot{W}}_{t}={\dot{m}}_{1}\cdot (h_{5}-h_{6s})\cdot \eta_{t}$$

6−1: Condensing process. Heat release of the working fluid is:

(16) $${\dot{Q}}_{C}={\dot{m}}_{1}(h_{6}-h_{1})$$

(17) $$\eta_{th}=\frac{{\dot{W}}_{t}-{\dot{W}}_{P1}-{\dot{W}}_{P2}}{{\dot{Q}}_{W}}=\frac{1/\phi \cdot (h_{5}-h_{6s})\cdot \eta_{t}-(h_{3}-h_{2})-1/\phi \cdot (h_{1^{\prime }}-h_{1})}{h_{4}-h_{3}}$$

**Figure 7:**
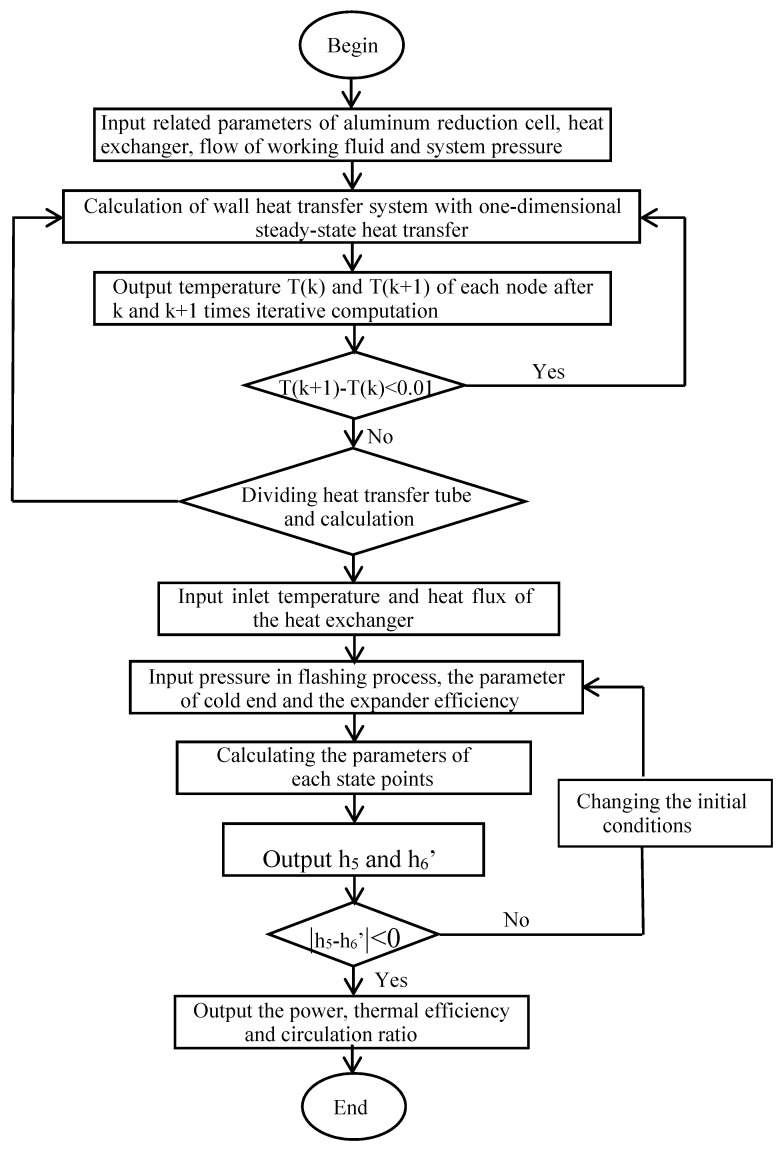
Flow chart of the calculation program.

According to the above calculation models, a thermodynamic calculation algorithm was coded by using Matlab software (MathWorks.Inc Natick, MA, USA), and the properties of R123 were calculated by REFPROP 8.0 software. The program chart is shown in Figure 7.

## 5. Results and Discussion

A 200 kA aluminum reduction cell was considered as the research object of this work. Usually, there are 18 ventilation spaces on each side of a cell, therefore, 36 sets of heat exchanger unit can be placed on it. The criteria conditions for calculating are as follows: R123 is selected as the working fluid. The pressure of the working fluid at heat exchanger outlet, called system pressure, is 2 MPa. The mass flow rate of the working fluid in each heat exchanger is 0.08 kg/s, and the efficiency of the expander is 0.75. The convection coefficient at the melt side is 550 W/(m^2^·K). The thickness of carbon layer, steel shell, insulating layer, and freeze ledge are 12, 1.2, 4, and 12 cm, respectively. The temperature of the electrolyte is 940 °C. The temperature of environment and cold water are 30 and 35 °C, respectively. The results below were obtained by changing a single variable in the criteria conditions (some results are related to one set of heat exchanger).

**Figure 8:**
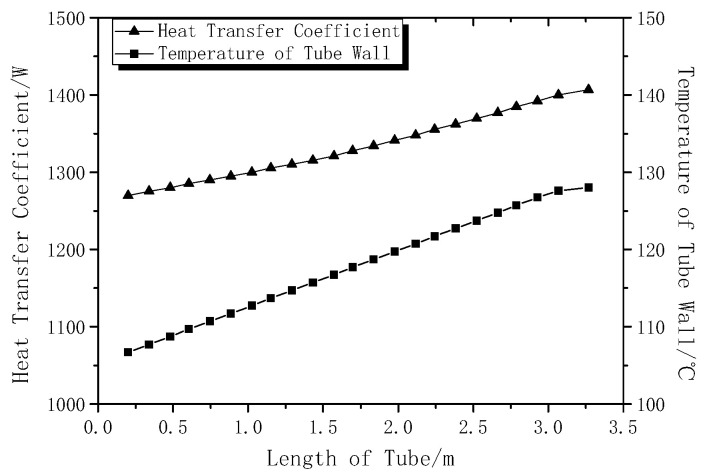
Heat transfer coefficient and wall temperature distribution in the tube.

The changing relationship of the convective coefficient and the wall temperature along the length of the tube are shown in Figure 8. It can be observed from the figure that the convective coefficient and the temperature of the wall increase along the tube length, almost linearly. The temperature of the wall changes from 107 to 128 °C.

**Figure 9:**
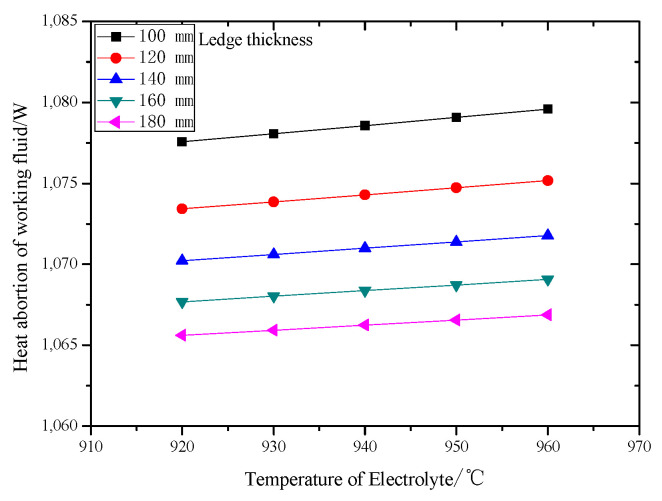
Relationship between heat absorption (one set of heat exchanger) of the working fluid and electrolyte temperature with various freeze ledge thicknesses.

The relationship between heat-absorption of the working fluid for a set of heat exchangers’ (the total amount of heat exchangers are 36) changes and electrolyte temperature is shown in Figure 9. Results show that under the same thickness of freeze ledge, the heat-absorbing capacity increases with the increasing electrolyte temperature, but the change is small. The heat-absorbing capacity of working fluid decreases with increasing of the thickness of the freeze ledge, when the electrolyte temperature is fixed, and this infers that heat transfer rate is mainly determined by the thermal resistance of cell wall layers, and it cannot be effectively increased by raising the cooling intensity of the heat exchanger.

**Figure 10:**
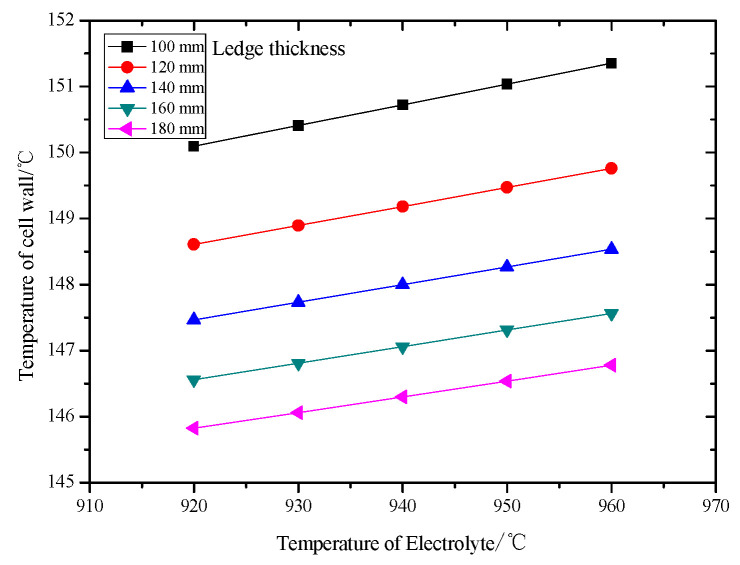
Temperature of cell wall at different electrolyte temperatures with various ledge thicknesses.

The relationship between the sidewall temperature and electrolyte temperature is discussed in Figure 10. It can be observed that the sidewall temperature increases with increasing electrolyte temperature. Also, the sidewall temperature is decreased with thicker freeze ledge under the same electrolyte temperature. The reason is that the thicker the freeze ledge, the greater the thermal resistance of the aluminum reduction cell’s side, so the sidewall temperature decreases. According to the calculation results, the maximum temperature of the sidewall is 150 °C, which is lower than that of normal operation.

**Figure 11:**
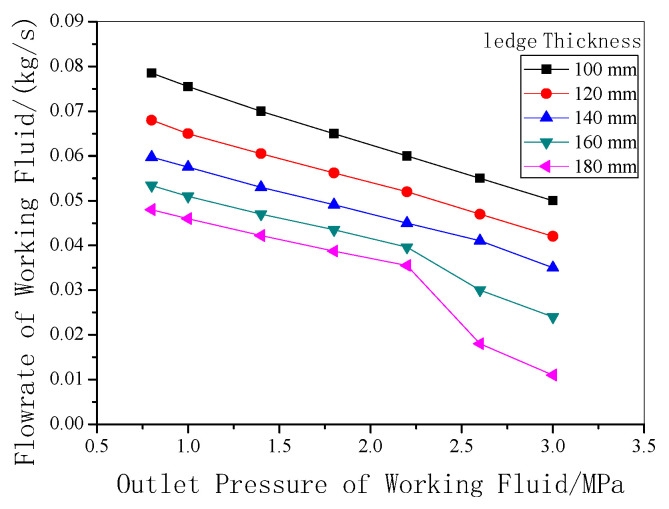
Flow rate of working fluid under different outlet pressures.

The relationship between the mass flow rate and system outlet pressure is discussed in Figure 11. It can be observed that the flow rate of working fluid decreases with increasing system pressure. Also, the flow rate is decreased with thicker freeze ledge under the same outlet pressure. The main reason is that the system pressure determines the temperature of working fluid, and the temperature of working fluid is directly related to the heat transfer rate.

**Figure 12:**
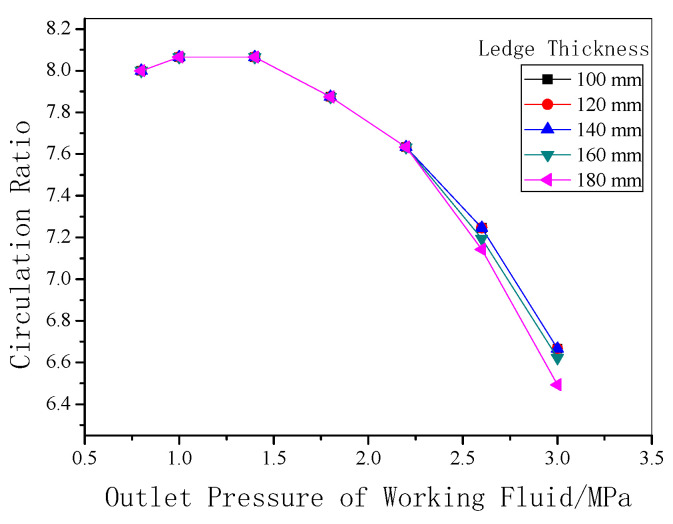
Working fluid circulation ratio under different outlet pressures.

As shown in Figure 12, the circulation ratio of working fluid in the flash evaporator is closely related to the system pressure. When the system pressure is above 1.5 MPa, with increasing system pressure, the circulation ratio slightly decreases. However, there is no evident influence from the thickness of freeze ledge on the circulation ratio.

**Figure 13:**
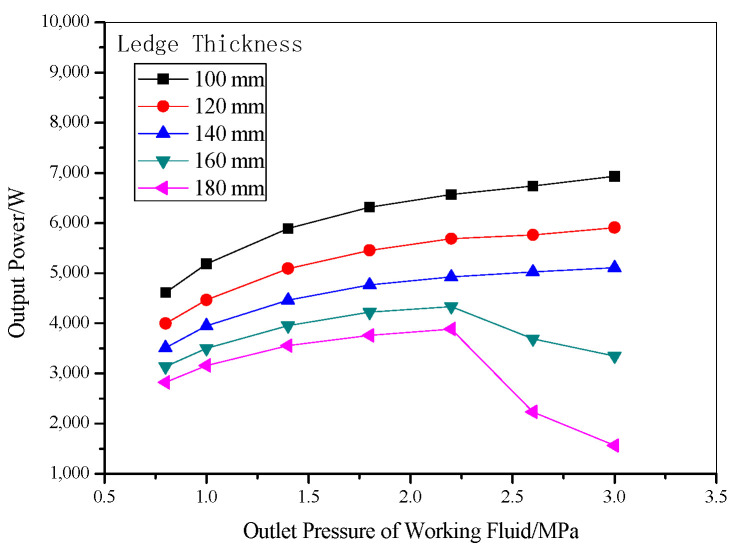
Output power under different outlet pressures with various ledge thicknesses.

Both output power and thermal efficiency are important indexes to evaluate the cycle performance. For a 200 kA electrolytic cell, the thirty-six heat exchanger units mentioned earlier can be set up. The relationship between the output power and the outlet pressure of the working fluid is shown in Figure 13. As displayed in the figure, with rising outlet pressure, the system output power increases. The reason is that higher output pressure can cause higher corresponding saturation temperature of the working fluid, resulting in a bigger enthalpy drop in the expander, and therefore, higher output power can be obtained. In addition, the output power of the system decreases with the thickness of freeze ledge as the heat transfer rate reduces. The output power can reach 5907 W when the system pressure is 3.0 MPa with 12 cm thickness of the freeze ledge. When the thickness of the ledge is increased to a certain value, the flow velocity of aluminum in the reduction cell decreases, which weakens the heat transfer between the electrolyte and the ledge, so when the thickness of the bath is increased to more than 160 mm and the pressure is greater than 2.5 MPa, the rate of flow and output work will be greatly reduced.

**Figure 14:**
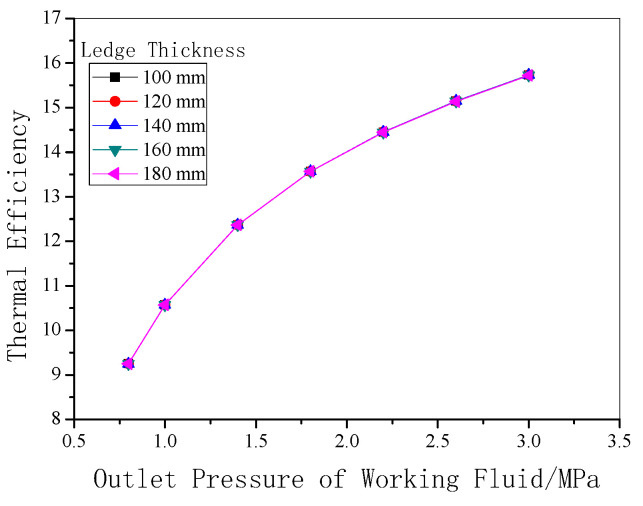
Thermal efficiency under different outlet pressures with various ledge thicknesses.

As shown in Figure 14, with rising outlet pressure, the thermal efficiency of the system increases. When the system pressure is 3.0 MPa, the thermal efficiency can reach 15.7%. Also, it is found that thermal efficiency of the system is not relevant to the thickness of the freeze ledge, which indicates that the change of heat transfer resistance, such as in the thickness of the freeze ledge, can affect the amount of heat exchanged and vary the output power proportionally at the same time.

Before studying the waste heat generation of the aluminum reduction cell’s sidewalls, we have made the recovery of the exhaust gas of the cell, in which R123 is the most suitable working fluid. In order to study the relationship of parameters, we chose the optimal working R123 closest to this system. By calculating the key parameters of power generation and thermal efficiency, the feasibility of waste heat recovery of sidewall was determined.

**Figure 15:**
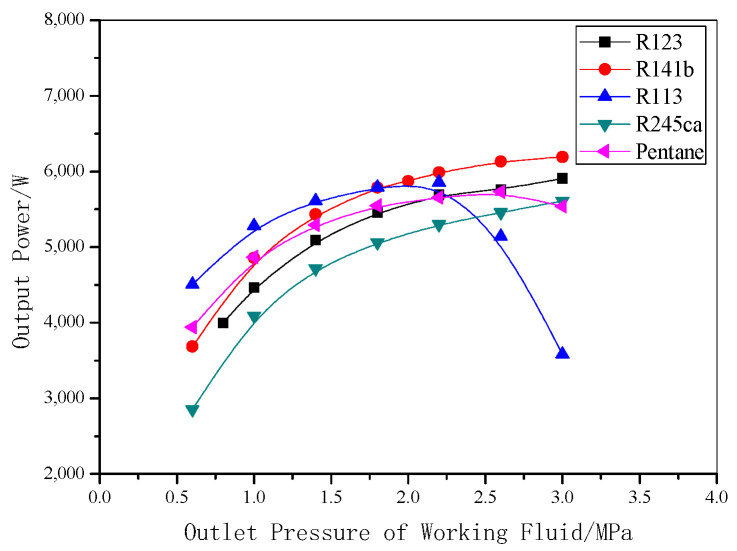
Output power under different outlet pressures for various working fluids.

**Figure 16:**
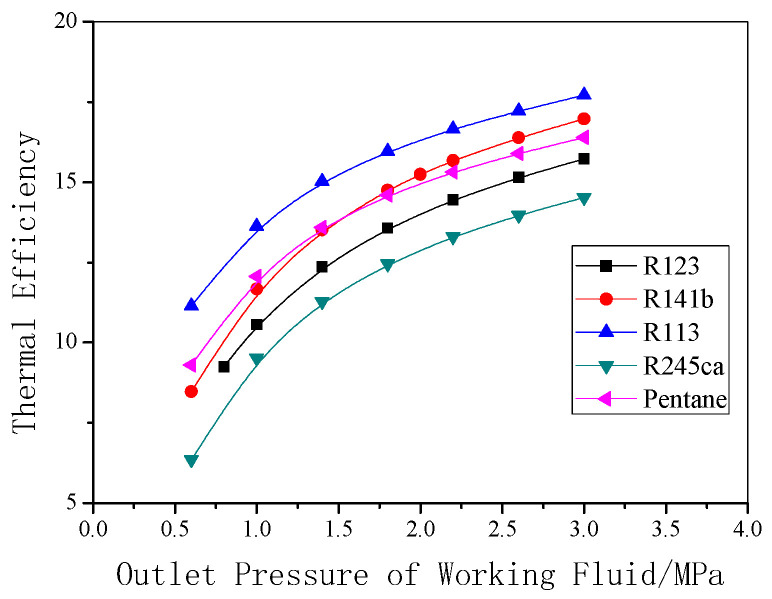
Thermal efficiency under different outlet pressures for various working fluids.

The calculated results above are related to R123. The following results are given with different working fluids. More than twenty possible working fluids were tested and calculated, and a few of them were selected because their cycle performances are relatively suitable. Calculations were conducted under the earlier criteria conditions, and the major results are shown in Figure 15 and Figure 16.

As observed in Figure 15, when different working fluid is used, there is a different relationship between the system output power and system pressure for an aluminum reduction cell of 200 kA series. For R123, R141b, R245ca, or pentane, with the increasing of system pressure, the output power of the system increases. When the system pressure is above 2.0 MPa, the system with R141b can generate the highest output power. Otherwise, the system with R113 will generate the highest output power. It can be concluded that if the system pressure is designed below 2.0 MPa, R113 should be the preferred working fluid, and when the designed system pressure is higher than 2.0 MPa, R141b should be the preferred working fluid.

In terms of cycle efficiency, as observed in Figure 16, pentane demonstrates a greater advantage, followed by R141b, and the cycle efficiency of R113 is the lowest, which means it is two different things to pursue the biggest output power and the highest cycle efficiency in design of the system.

## 6. Conclusions

According to the thermal dissipation characteristics on the sidewalls of aluminum reduction cells, a WHPGS with flash cycle with organic working fluids was proposed. Thermal performance analysis on this system was conducted, and conclusions were made as follows:

(1) After the heat exchange system is added to the sidewalls of aluminum reduction cells, the thermal resistance of walls becomes the major factor determining the heat absorption of working fluid, and therefore, the heat transfer rate cannot be increased by raising cooling intensity.
(2) The heat transfer rate can be increased by reducing the thickness of the cell or raising electrolyte temperature.
(3) For an aluminum reduction cell of 200 kA series, the output power of the ORC system can reach 5907 W and the cycle efficiency can reach 15.7% when R123 is used as the working fluid, and the electrolyte temperature, the thickness of the freeze ledge, and the system pressure are 940 °C, 12 cm, and 3.0 MPa, respectively.
(4) The output power of the system varies with different working fluid selected. If the designed system pressure is lower than 2.0 MPa, R113 is the preferred working fluid, and when the pressure is higher than 2.0 MPa, R141b is the preferred working fluid. As an example, when the designed system pressure is 3.0 MPa, and R141b is chosen as the working fluid, the output power from such a system based on an aluminum electrolytic cell can reach 6190 W and the thermal efficiency can reach 17.7%. As a reference, a 200 kA electrolysis series with an annual output of 100 kilotons has 200 cells. The power generation system installed in this electrolysis series can provide 1200 kW installed power generation capacity.

## Figures and Tables

**Table 1:** Parameters related to a wall heat exchanger.

Parameters	Value
General dimension (W × H × T)	400 × 650 × 20 mm
Heat-transfer tube	Φ10 × 1 mm
Efficient heat transfer area	0.12 m^2^
Designed pressure	<3.0 MPa
Flow velocity of working fluid	<3 m/s

## Abbreviations

ORC	Organic Rankine Cycle
WHPGS	waste heat power generation system

## Symbols

*B*	thickness of every layer, mm
*C_pi_*	specific heat capacity of the working fluid, J kg^−1^ K^−1^
*d*	diameter of the tube, mm
*h*	convective coefficient, W m^−2^ K^−1^
*i*, *j*, *k*, *l*, *m*, *n*, *p*, *q*, *r*, *s*	number of temperature node
*l*	length of every section of the tube, mm
$\dot{m}$	mass flowrate, kg/s
Nu	Nusselt number
Pr	Prandtl number
$\dot{Q}$	heat, W
$\dot{q}$	heat flux, W m^−2^
*R*	thermal resistance, m^2^ K W^−1^
Re	Reynolds number
*T*	temperature, K °C
*t*_*i*−1_	inlet temperature, °C
*t_i_*	outlet temperature, °C
$\dot{W}$	work, W
${\dot{W}}_{p}$	work of the pump, W

## Greek Symbols

λ	thermal conductivity, W m^−1^ K^−1^
ϕ	circulating rate
η	efficiency of the expander

## Subscripts

A	alumina
air	environment side
Ca	carbon block
Co	copper tube
C	Condensing process
C1	contact resistance layer between carbon block and steel layer
C2	contact resistance layer between steel layer and inner layer of heat-transfer from inner freeze to working fluid
exi	working fluid side
Fr	freeze layer
f	working fluid
ins	insulating layer
M	electrolyte side
out	from working fluid to circumstance
P	pump
St	steel layer
t	expander
th	thermal
